# PrEP risk perception and adherence among men who have sex with men: a prospective cohort study based on growth mixture model

**DOI:** 10.1186/s12879-022-07966-3

**Published:** 2022-12-30

**Authors:** Bing Lin, Jiaxiu Liu, Xiaoni Zhong

**Affiliations:** 1 School of Public Health, Chongqing Medical University, Chongqing, China; 2 Research Center for Medicine and Social Development, Number One Medical College Road, Yuzhong District, Chongqing, 400010 China; 3 School of Medical Informatics, Chongqing Medical University, Chongqing, China

**Keywords:** Pre-exposure prophylaxis, Risk perception, Men who have sex with men, Adherence, Growth mixture model

## Abstract

**Background:**

It can be considered that pre-exposure prophylaxis (PrEP) risk perception is the negative experiences or concerns about taking PrEP. The aim of this study is to explore the longitudinal trajectory of PrEP risk perception among men who have sex with men (MSM) and its impact on adherence.

**Methods:**

Data related to PrEP risk perception and adherence were derived from a prospective cohort study in Western China from 2013 to 2015. Subjects were categorized into the time-driven, event-driven and blank control groups. Tenofovir disoproxil fumarate (TDF) was administered to subjects in the time-driven and event-driven groups, and all subjects were followed up every 12 weeks. The PrEP risk perception scale was constructed, and the growth mixture model (GMM) was used to classify longitudinal PrEP risk perception. The effect of different levels of PrEP risk perception on drug adherence was explored using generalized estimating equations (GEE) with relative risk (RR) and 95% confidence interval (CI).

**Results:**

The PrEP risk perception scale consists of 4 dimensions and 16 items with Cronbach's alpha = 0.828 and a good model fit. According to the GMM analysis, the subjects' PrEP risk perceptions were separated into two groups: a "high-risk perception group" (n = 133) and a "low-risk perception group" (n = 493), where the proportion of high levels of drug adherence were 57.89% and 68.35%, respectively (p = 0.024). High levels of PrEP risk perception in the MSM population hinder drug adherence (RR = 0.71, 95% CI 0.50 to 0.99, p = 0.046). The results of this study were validated in the subsequent PrEP projects conducted in 2019 to 2021.

**Conclusion:**

This study demonstrates that high levels of PrEP risk perception in the MSM population are an obstacle to drug adherence, emphasizing the necessity of focusing on PrEP risk perception in this population and the value of its application in the current context.

**Supplementary Information:**

The online version contains supplementary material available at 10.1186/s12879-022-07966-3.

## Background

In 2014, the Joint United Nations Program on HIV and AIDS (UNAIDS) established the "90-90-90" target for human immunodeficiency virus (HIV) prevention and treatment. However, several studies show that we are still a long way from meeting the "90-90-90" and "2030" targets [1, 2]. To date, acquired immune deficiency syndrome (AIDS) related deaths are on track to decline globally, but the rate of decline in new HIV infections is falling short of the anticipated goal [3]. As a result, strengthening HIV prevention and control measures are critical.

As a high-risk group for HIV transmission, the population of men who have sex with men (MSM) poses a significant challenge to the global HIV response. The global MSM population is projected to be around 20 times more likely than the general population to be infected with HIV [4]. Pre-exposure prophylaxis (PrEP) is a biological HIV prevention intervention that focuses on reducing the risk of HIV infection through daily (or event-based) oral antiretroviral medication [5]. Oral PrEP has been shown to be an effective strategy to prevent HIV infection in high-risk populations [6]. Meanwhile, a multicenter study in the United States and a qualitative descriptive study in Africa both demonstrate that the PrEP strategy is feasibility and acceptability among MSM population [7, 8]. However, the efficacy of PrEP is highly dependent on adherence [9, 10]. When the adherence is high, the risk of HIV infection can be significantly reduced [11]. As a consequence, adherence has an essential role in PrEP prevention strategies.

Risk is mostly associated with negative events or outcomes such as injury, damage and loss. It refers to the likelihood of an undesirable event occurring and the magnitude of the loss it causes within a given event or environment [12]. With the development of society and technology, people are facing more and more risks, and the scales and characteristics of risks have become complex and diverse. Moreover, risk perception is the subjective feeling of people about their own risk and is an important factor influencing behavior [13]. Individuals with high levels of risk perception tend to avoid risks when faced with potential threats, thus changing relevant behavioral measures to reduce their losses [14]. Risks are ubiquitous and closely linked to the consequences of individual's decisions and behaviors. Therefore, risk perception requires extensive attention. We assume that when MSM start using PrEP it may make them feel like it's a risky thing to do, which can lead to negative experiences and concerns. For example, fear of privacy disclosure, accusations of promiscuity [15], being mistaken for HIV-positive, fear of drug side effects and drug efficacy [16]. It is this uncertainty and complexity in the PrEP process that gives rise to their risk perception. However, there is no clear definition of PrEP risk perception currently. Therefore, we define "PrEP risk perception" as the risks (negative experiences and concerns) that subjects perceived during PrEP. It can be thought of as the user's awareness, perception and understanding of the risk factors and risk characteristics of the PrEP process. If the subjects are overly concerned in the PrEP process, this may affect their willingness and motivation to engage in PrEP, which will reduce its efficacy.

Since risk perception is a critical variable in explaining individual behavior, at the same time, adherence is an important factor in the efficacy of PrEP, an in-depth understanding of PrEP risk perception and its impact on adherence in the MSM population will help to explain the relationship between risk perception and adherence.

Most measures of risk perception use questionnaire that ask subjects to assess several characteristics of potential risks. This approach is known as the psychometric paradigm. For example, two Chinese scholars constructed a 7-point Likert scale to assess participants' perceived likelihood of developing breast cancer and the degree of serious consequences [17]. Other researchers have investigated whether risk perception motivates preventive behavior by asking subjects to response eight perceived risk items, which were scored correct/incorrect and summed [18]. Previous study also systematically assessed risk perception in four areas: "outcome uncertainty", "potential gains and losses", "situational framing", and "personal expectations" [19]. As can be seen from the studies above, earlier researchers primarily employed questionnaires or scales to assess risk perception, which has the advantages of simplicity and low cost. As a result, our study aimed to develop a PrEP risk perception scale based on the theoretical basis of previous studies and the various dimensions that influence risk perception.

The majority of risk perception studies are now cross-sectional. Few studies have looked into the PrEP risk perception, and even fewer have explored the longitudinal relationship between PrEP risk perception and adherence. We established data based on a prospective cohort study in Western China from 2013 to 2015 and developed the PrEP risk perception scale. The growth mixture model (GMM) and generalized estimating equations (GEE) were used to investigate the effect of PrEP risk perception on adherence, providing a theoretical foundation for future research on “risk perception-adherence”. Meanwhile, the study of PrEP risk perception reflects the acceptability and feasibility of PrEP among MSM. An in-depth understanding of PrEP risk perception can help researchers come up with new ways to promote prevention in this population.

## Methods

### Participants and procedures

This study was a prospective cohort study in Western China from 2013 to 2015 (registration number: ChiCTR-TRC-13003849, date of registration: 24/06/2013). Non-probability sampling strategies, such as collaboration with non-governmental organizations (NGOs), posting information on the MSM website, and snowball sampling, were used to recruit participants from four regions in Western China: Chongqing, Sichuan, Xinjiang, and Guangxi. The inclusion criteria in the clinical trial were as follows: (1) biological men (the sex is male when they were born) (2) signed informed consent; (3) age ≥ 18 years old and ≤ 65 years old; (4) negative in HIV antibody and P24 antigen test; (5) at least one or more same-sex partners prior to the trial; (6) willingness to use drug under supervision and to comply with follow-up arrangements.

The eligible subjects were randomly divided into time-driven, event-driven and blank control groups. The blank control group did not take any medicines only included in the cohort management. The time-driven group was given oral daily Tenofovir disoproxil fumarate (TDF, 300 mg/tablet). Subjects in the event-driven group took 300 mg TDF orally 48–24 h before sexual activity, and 300 mg TDF 2 h after sexual activity. The dosage was no more than 300 mg within 24 h. The time-driven group and event-driven group were collectively referred to as the medicine group. After enrollment, subjects were followed face-to-face every 12 weeks to complete a new round of follow-up questionnaires, and medicines were dispensed.

### Measures

The baseline questionnaire mainly included: demographic characteristics and HIV-related characteristics. Demographic characteristics mainly included: age, household register location, ethnic, education attainment, marital status, employment status and monthly personal income. HIV-related characteristics mainly included: HIV knowledge, HIV counseling, HIV testing, sexual role and female sexual partners. The HIV knowledge score is made up of 13 HIV-related questions. Higher scores indicated that the participant knew more information about HIV. We define an HIV knowledge score of ≥ 11 as "high" [20, 21]. Sexual role is the way of having penetrative sex with a male partner, such as the inserter (like “top”) and the receiver (like “bottle”).

The follow-up questionnaire included: drug adherence, high-risk anal sex, number of sexual partners, and the PrEP risk perception scale. Adherence was measured by self-report. Subjects were asked, "In the last 2 weeks, did you miss any doses? How many times did you miss?" The subjects should answer “yes” or “no” as well as how many times they miss to take medicine. Adherence equals 100% - (number of times subjects forget to take a medicine/number of times subjects should take a medicine). The number of times that the time-driven group should take a medicine is the number of days (14 days); the number of times that the event-driven group should take a medication is twice number of sex act. If an illogical response occurs, participants will be reminded to answer the question again at the study site. Adherence ≥ 80% defined as "high-level" [20, 22, 23]. High-risk anal sex was defined as not using a condom during anal sex. Subjects were asked about the number of anal sex acts and the number of times a condom was used during sex.

### The PrEP risk perception scale

**Fig. 1:**
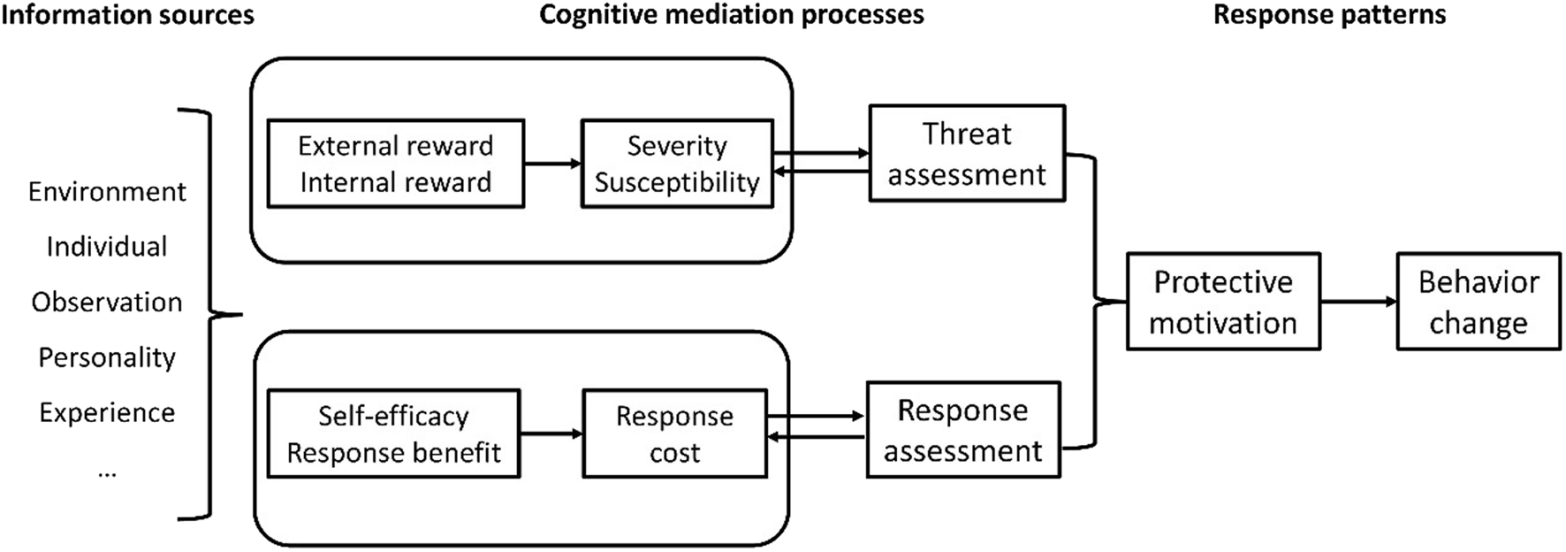
Structural framework diagram of the Protective Motivation Theory (PMT)

According to the Protective Motivation Theory (PMT) [24], the pattern of behavior formation, can be divided into three parts, which are information sources, cognitive mediation processes, and response patterns (Fig. 1). This theory suggests that information about the threat posed by an individual's relevant behavior triggers two cognitive processes, threat assessment and response assessment, which work together to form protective motivation and subsequently promote or inhibit the occurrence of behavior (behavior change). Numerous studies have shown that the theory is a model of behavior change and prediction applicable to Chinese cultural contexts [25, 26]. Also, recent studies have applied the PMT to the study of adherence in MSM population. The results show that improving threat perception, controlling and reducing response costs can effectively improve PrEP adherence [27]. Therefore, the theory can be used as a reference for MSM population in the study of adherence. In the early stage of the project, we conducted qualitative interviews with a portion of MSM. By summarizing the contents of the interviews and combining with the PMT, we provided a theoretical basis for constructing the PrEP risk perception scale.

PrEP risk perception refers to the negative experiences or concerns about taking PrEP, and the scale is composed of four dimensions: "Personal experience and self-efficacy", "Potential concerns", "Medicine impacts" and "Medical mistrust". "Personal experience and self-efficacy" refers to the relief from fear/risk of AIDS and a sense of confidence in taking medicine. "Potential concerns" refers to the potential concerns due to use PrEP. "Medicine impacts" refers to the negative experiences associated with drugs during PrEP. "Medical mistrust" refers to mistrust of doctors when taking medicine.

Both Exploratory Factor Analysis (EFA) and Confirmatory Factor Analysis (CFA) of the PrEP risk perception scale were implemented using Mplus software. The root mean-square error of approximation (RMSEA), comparative fit index (CFI), Tucker-Lewis index (TLI), standardized root mean squared residual (SRMR) were used to assess model fit. We used conventional cutoff criteria indicating an acceptable model fit that RMSEA and SRMR < 0.1, CFI and TLI > 0.8.

We conducted a preliminary Exploratory Factor Analysis to test the four dimensions. Then Cronbach's alpha was calculated after adjusting and deleting the items in the scale, and assessing the reliability and acceptable internal consistency reliability of each factor. “Personal experience and self-efficacy” (Cronbach's alpha = 0.812), “Potential concerns” (Cronbach's alpha = 0.812), “Medicine impacts” (Cronbach's alpha = 0.756), and “Medical mistrust” (Cronbach's alpha = 0.884) are all found to be significant. The specific descriptive statistics of the four dimensions are presented in Table 1. Based on the results: the 5-point Likert PrEP risk perception scale had a total of 16 items (Cronbach's alpha = 0.828). “1” indicates “completely no”, and “5” indicates “always”. The scale has a minimum score of 16 and a maximum score of 80. A higher score means a more negative experience or concern in taking PrEP. For instance, experiencing more concerns of judgment, feeling less confident and perceiving less support from doctors to take PrEP. Finally, a Confirmatory Factor Analysis was conducted: Chi-square = 587.652, df = 98, RMSEA = 0.080, CFI = 0.897, TLI = 0.874, and SRMR = 0.058. The model fit was good, indicating the feasibility of the scale.

**Table 1:** Exploratory factor analysis of the PrEP risk perception scale

Factors	Items	Range	Geomin rotated loadings
Personal experience and self-efficacy	I think medicine make me safe, away from AIDS^a^	1–5	0.727
	I can remember to take my medicine on time^a^	1–5	0.703
	My fear of AIDS is lessened^a^	1–5	0.640
	I'm used to taking medicine^a^	1–5	0.670
			Cronbach's alpha = 0.812
Potential concerns	I worry that the medicine has no effect	1–5	0.817
	I worry about the side effects of medicine	1–5	0.801
	I'm worried that homosexual partners know I'm taking medicine	1–5	0.654
	I worry that other people will discriminate me when they know I am on medicine	1–5	0.624
	I felt the side effects of the medicine	1–5	0.303
	I find it inconvenient to take the medicine	1–5	0.495
	I think it’s very troublesome to take the medicine	1–5	0.484
			Cronbach's alpha = 0.812
Medicine impacts	I find it difficult to swallow medicine	1–5	0.644
	The smell of medicine makes me feel uncomfortable	1–5	0.921
	The dosage form of medicine I do not like to take	1–5	0.437
			Cronbach's alpha = 0.756
Medical mistrust	I think the doctors here discriminate against me^a^	1–5	0.952
	I do not trust the doctors here^a^	1–5	0.818
			Cronbach's alpha = 0.884
Composite score		16–80	Cronbach's alpha = 0.828

^a^Indicates reverse items

AIDS: Acquired Immune Deficiency Syndrome

Model fit information of Exploratory Factor Analysis: Chi-square = 251.657, df = 62, RMSEA = 0.062, CFI = 0.946, TLI = 0.896, SRMR = 0.026

### Growth mixture model (GMM)

The growth mixture model (GMM) was employed to investigate the issue of group heterogeneity in this study. The model divides the group into potential categories, describing the developmental trajectory of each category separately, and individuals in each category have similar or identical trajectories [28, 29]. We assume that the risk perception was divided into several potential categories during the PrEP process, and we utilize GMM to identify the potential categories, and estimate the trajectory of different potential categories. The GMM was implemented using Mplus software. The model fitting criteria were as follows: Akaike (AIC), Bayesian (BIC), and Sample-Size Adjusted BIC (aBIC). The smaller these three indictors are, the better the model match. Vuong-Lo-Mendell-Rubin Likelihood Ratio Test (VLRT) and Bootstrapped Likelihood Ratio Test (BLRT) should have p-values of less than 0.05 and Entropy higher than 0.70.

### Statistical analysis

**Fig. 2:**
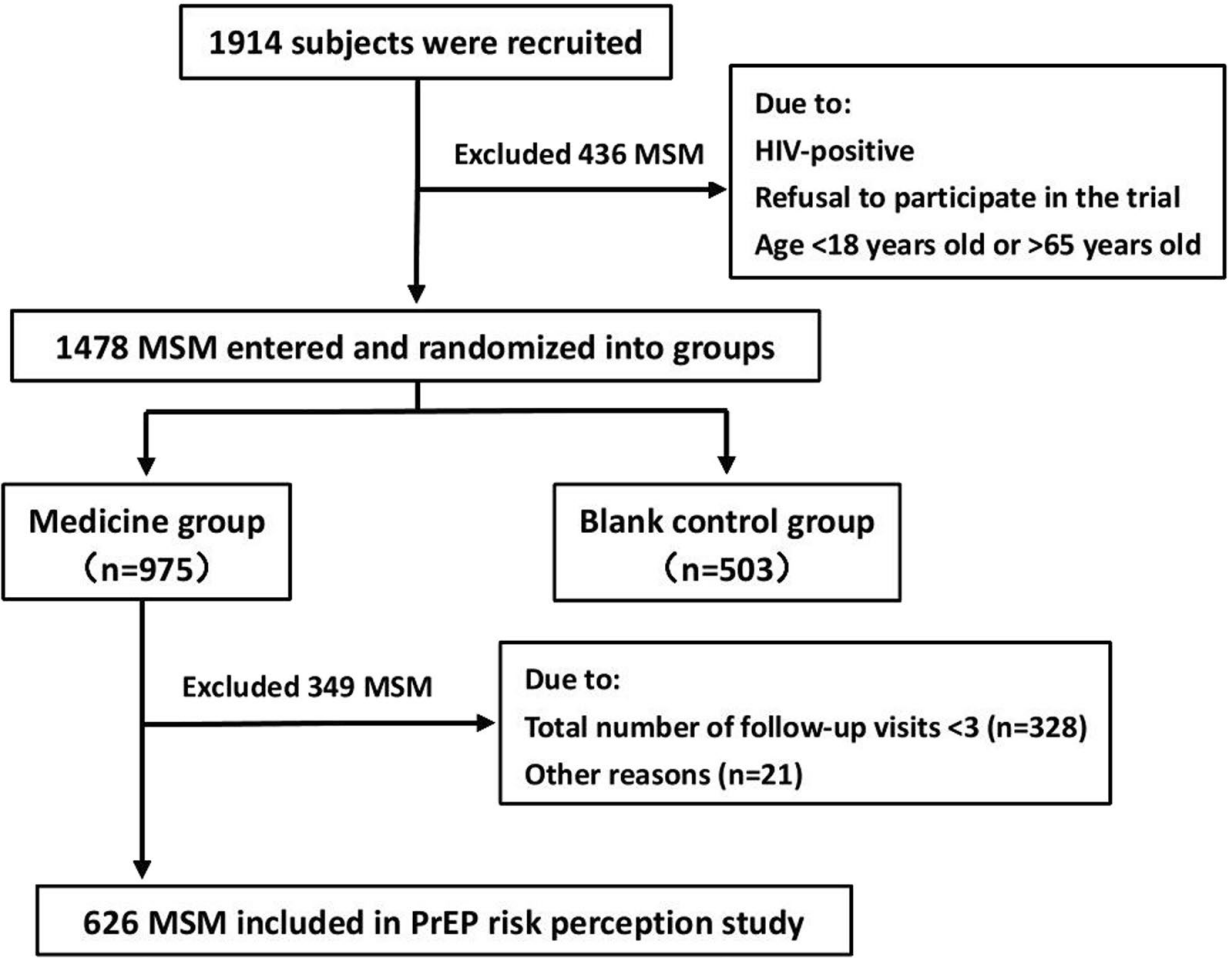
Flow chart f of participants’ enrollments. MSM: men who have sex with men; HIV: human immunodeficiency virus

We analyzed data from the prospective cohort study in the medicine group. Subjects will not be included in the data analysis if they participated in the total number of follow-up visits < 3 due to the need to establish longitudinal data on PrEP risk perception. A total of 626 MSM were finally included in the PrEP risk perception study. The study population screening process is shown in Fig. 2. On the basis of the fitting results of the growth mixture model (GMM), longitudinal trajectories of PrEP risk perception were developed, and subgroups of latent categories that satisfied the criteria were screened. The average value of adherence of each measurement was taken to compare the difference among different latent risk perception groups. Generalized estimating equations (GEE) were used to investigate the effects of varying levels of PrEP risk perception on drug adherence with relative risk (RR) and 95% confidence interval (CI) after correcting for other factors. p < 0.05 represents statistical significance. SAS software was used for analysis.

## Results

**Fig. 3:**
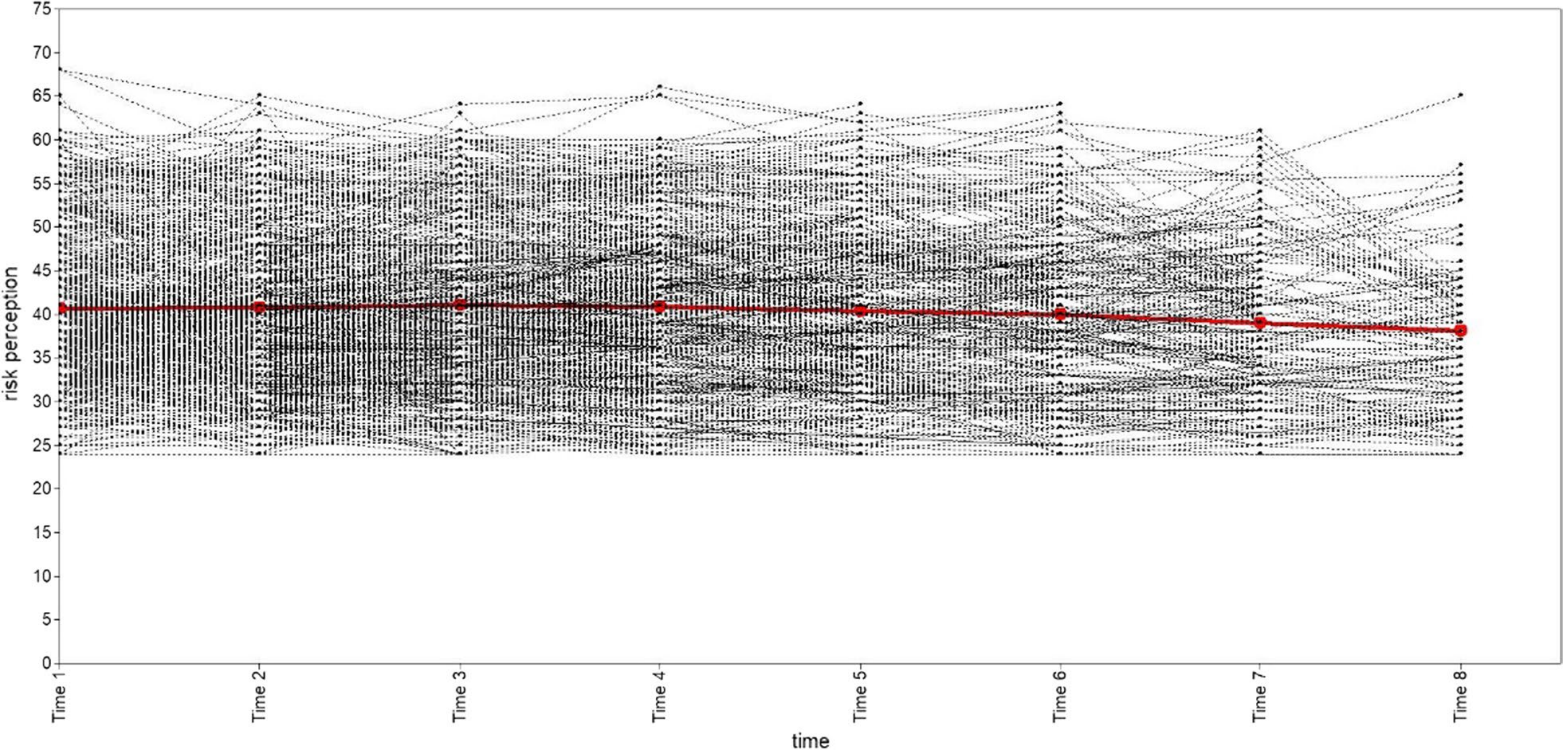
Trajectory analysis plot of PrEP risk perception one category in MSM population. The horizontal coordinate is the time of follow-up, and the vertical coordinate is the score of the PrEP risk perception

**Fig. 4:**
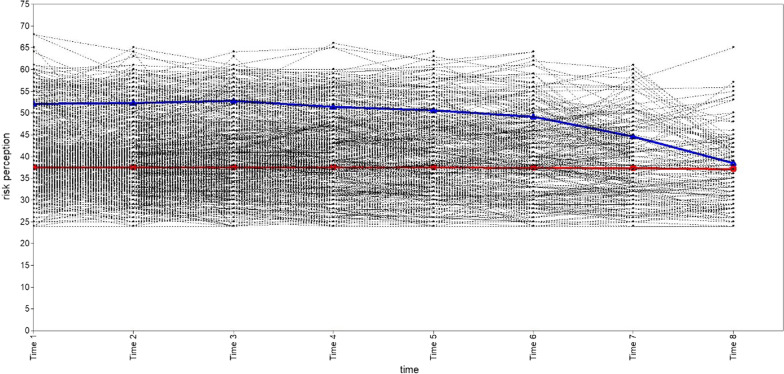
Trajectory analysis plot of PrEP risk perception two categories in MSM population (we selected). The horizontal coordinate is the time of follow-up, and the vertical coordinate is the score of the PrEP risk perception. The red line represents “Category 1” (n = 493, 78.75%), with a low initial value of PrEP risk perception and a smoother overall level. We named “Category 1” as the “low-risk perception group”. The blue line represents “Category 2” (n = 133, 21.25%), with higher initial values of PrEP risk perception and a gradual decrease over time. We named “Category 2” as the “high-risk perception group”

**Fig. 5:**
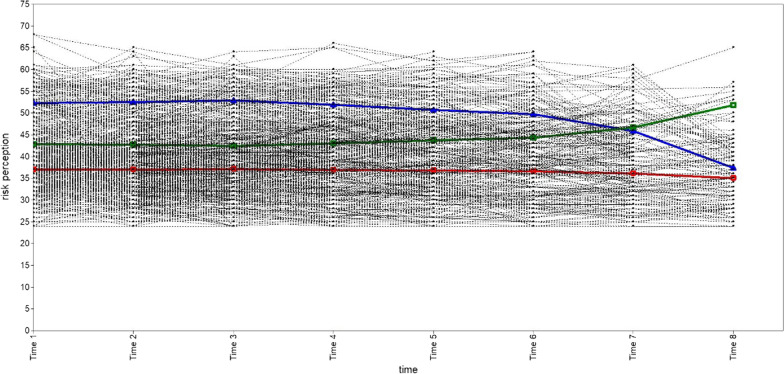
Trajectory analysis plot of PrEP risk perception three categories in MSM population. The horizontal coordinate is the time of follow-up, and the vertical coordinate is the score of the PrEP risk perception. The red line represents “Category 1” (n = 477, 76.20%); the blue line represents “Category 2” (n = 137, 21.89%); the green line represents “Category 3” (n = 12, 1.91%)

**Fig. 6:**
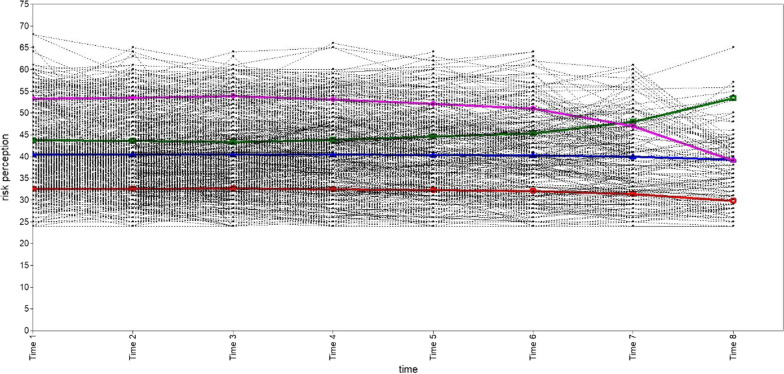
Trajectory analysis plot of PrEP risk perception four categories in MSM population. The horizontal coordinate is the time of follow-up, and the vertical coordinate is the score of the PrEP risk perception. The red line represents “Category 1” (n = 178, 27.43%); the blue line represents “Category 2” (n = 315, 50.32%); the green line represents “Category 3” (n = 11, 1.76%); the purple line represents “Category 4” (n = 122, 19.49%)

We explored one, two, three, and four categories of subgroups for PrEP risk perception in the MSM population. A summary of the GMM model fit information for each category is shown in Table 2. According to the best model fit criteria, the GMM model with two classifications had the largest Entropy value and the most reasonable proportion of classifications, even if its AIC, BIC and aBIC values were not the least, and both VLRT and BLRT were statistically significant. Therefore, the two-category GMM model is recognized as the best. The number of "Category 1" is 493 (78.75%), and the number of "Category 2" is 133 (21.25%). The trend of GMM trajectories for each category is shown in Figs. 3, 4, 5, 6. The figure illustrates that "Category 2" has a high level of PrEP risk perception at the early stage, and then decreases over time. "Category 1", on the other hand, is overall smooth and has a lower PrEP risk perception. Therefore, we named "Category 1" (n = 493) as the "low-risk perception group" and "Category 2" (n = 133) as the "high-risk perception group" in the two-category GMM model.

**Table 2:** Growth mixture model (GMM) model classification fit information for each latent category

Category	AIC	BIC	aBIC	Entropy	VLRT	BLRT	Category probability
1C	19,832.575	19,912.483	19,855.335	–	–	–	1
2C	19,753.029	19,846.255	19,779.583	0.780	< 0.001	< 0.001	0.7875/0.2125
3C	19,731.666	19,838.210	19,762.013	0.689	0.033	< 0.001	0.7620/0.2189/0.0191
4C	19,710.798	19,830.660	19,744.939	0.660	0.039	< 0.001	0.2843/0.5032/0.0176/0.1949

AIC: Akaike; BIC: Bayesian; aBIC: Sample-Size Adjusted BIC; VLRT: Vuong-Lo-Mendell-Rubin Likelihood Ratio Test; BLRT: Bootstrapped Likelihood Ratio Test

The GMM model parameter metrics for both categories are shown in Table 3. The intercept factor (I) and slope factor (S) variance estimates were 24.599 and 0.003, respectively, with p < 0.0001 for the intercept factor and p = 0.674 for the slope factor, indicating that the initial values of PrEP risk perceptions differed between individuals in the two categories, while the rate of PrEP risk perception growth was not statistically different between individuals. The correlation coefficient between the intercept growth factor and the slope growth factor was − 0.134 (p = 0.403), suggesting no significant correlation between the rate of change in growth and the initial state.

**Table 3:** Indicators of the growth mixture model (GMM) with two categories

Variables			Estimate	SE	Est/SE	p-value
Category 1	Mean	I	37.494	0.325	115.334	< 0.001
		S	0.007	0.011	0.590	0.555
Category 2	Mean	I	52.090	0.787	66.223	< 0.001
		S	0.241	0.278	0.868	0.386
Category 1 vs. Category 2	Variance	I	24.599	2.223	11.064	< 0.001
		S	0.003	0.007	0.421	0.674
		S With I	− 0.134	0.160	− 0.836	0.403

I: intercept; S: slope; SE: standard error; Est: estimate

We described demographic and HIV-related characteristics for both groups. At the same time, the average value of multiple measurement adherence was taken to compare the difference in the proportion of high adherence between the two groups. According to the Chi-square test results, the high adherence proportions of low-risk perception group and high-risk perception group were 68.35% and 57.89% respectively (χ^2^ = 5.12, p = 0.024), which suggested that the high level of PrEP risk perception had a significant effect on drug adherence and further analysis was required (Table 4).

**Table 4:** Descriptive analysis of demographic and HIV-related characteristics and drug adherence in low-risk perception group and high-risk perception group

Variables	All N = 626(%)	Low-risk perception group N = 493(%)	High-risk perception group N = 133(%)
*Demographic characteristics*			
Age			
Median (IQR)	29 (24 ~ 36)	29 (24 ~ 36)	29 (24 ~ 34)
18 ~ 30	335 (53.51)	265 (53.75)	70 (52.63)
30 ~ 45	252 (40.26)	195 (31.15)	57 (42.86)
≥ 45	39 (6.23)	33 (6.70)	6 (4.51)
Household register location			
Urban	483 (77.16)	372 (75.46)	111 (83.46)
Rural	143 (22.84)	121 (24.54)	22 (16.54)
Ethnic			
Ethnic Han	558 (89.14)	448 (90.87)	110 (83.71)
Ethnic minorities	68 (10.86)	45 (9.13)	23 (17.29)
Education attainment			
Junior high and below	14 (2.24)	11 (2.23)	3 (2.26)
High school	56 (8.95)	45 (9.13)	11 (8.27)
College	155 (24.76)	134 (27.18)	21 (15.79)
Undergraduate and higher	401 (64.05)	303 (61.46)	98 (73.68)
Marital status			
Single	527 (84.19)	414 (83.98)	113 (84.86)
Married	99 (15.81)	79 (16.02)	20 (15.04)
Employment status^a^			
Employed	504 (80.64)	393 (79.72)	111 (84.09)
Internal student	74 (11.84)	59 (11.97)	15 (11.36)
Jobless/Retirement	47 (7.52)	41 (8.31)	6 (4.55)
Monthly personal income^a^			
< 3000 CNY	315 (50.97)	273 (55.83)	42 (32.56)
3000 ~ 10,000 CNY	288 (46.60)	204 (41.72)	84 (65.12)
≥ 10,000 CNY	15(2.43)	12 (2.45)	3 (2.32)
*HIV-related characteristics*			
HIV knowledge score			
≥ 11	256 (40.89)	206 (41.78)	50 (37.59)
< 11	370 (59.11)	287 (58.22)	83 (62.41)
Have you been tested for HIV before this time^a^			
Yes	527 (84.32)	402 (81.71)	125 (93.98)
No	98 (15.68)	90 (18.29)	8 (6.02)
Have you ever received HIV counseling before this time^a^			
Yes	430 (69.13)	330 (67.48)	100 (75.19)
No	192 (30.87)	159 (32.52)	33 (24.81)
Sexual role during sexual intercourse with a male partner^a^			
Bottle	93 (16.64)	77 (17.54)	16 (13.33)
Both	157 (28.09)	126 (28.70)	31 (25.83)
Top	309 (55.27)	236 (53.76)	73 (60.84)
Have you had a female sexual partner in the last 6 months (steady and casual)^a^			
No	488 (85.31)	388 (85.84)	100 (83.33)
Yes	84 (14.69)	64 (14.16)	20 (16.67)
Drug adherence^b^			
Adherence			
High (≥ 80%)	414 (66.13)	337 (68.35)	77 (57.89)
Low (< 80%)	212 (33.87)	156 (31.65)	56 (42.11)
Medicine group			
Time-driven group	368 (58.79)	281 (57.00)	87 (65.41)
Event-driven group	258 (41.21)	212 (43.00)	46 (34.59)

^a^Indicates missing data

^b^Drug adherence was measured longitudinally, where adherence was averaged over multiple measurements

IQR: inter quartile range; HIV: human immunodeficiency virus. CNY: Chinese Yuan Renminbi

Generalized estimating equations (GEE) were utilized to explore the effect of different levels of PrEP risk perception on adherence by incorporating demographic and HIV-related characteristics (Table 5). Finally, we obtained that adherence was higher in the event-driven group. Based on RR values, the probability of high adherence occurred in the event-driven group was 1.31 times higher (95% CI 1.00 to 1.70) than in the time-driven group and was statistically significant (p = 0.049). PrEP risk perception was one of the influencing factors for drug adherence (p = 0.046). The higher the PrEP risk perception in MSM population is, the lower their drug adherence become (RR = 0.71, 95% CI 0.50 to 0.99). Also, high HIV knowledge scores had a positive effect on adherence, and the probability of high adherence occurred in high-level group was 1.38 times higher (95% CI 1.04 to 1.83) than in low-level group and was statistically significant (p = 0.025). MSM who had high-risk anal sex exhibited reduced adherence compared to the controls (RR = 0.70, 95% CI 0.54 to 0.89, p = 0.004).

**Table 5:** Generalized estimating equations (GEE) analysis affecting PrEP medication adherence

Variables	β (95% CI)	Z	RR (95% CI)	χ^2^	p-value
Medicine group (Event-driven vs. Time-driven)	0.27 (0.01 to 0.53)	1.97	1.31 (1.00 to 1.70)	3.87	0.049
HIV knowledge scores (High-level vs. Low-level)	0.32 (0.04 to 0.60)	2.24	1.38(1.04 to 1.83)	5.03	0.025
High-risk anal sex (Yes vs. No)	− 0.36 (− 0.61 to − 0.12)	− 2.88	0.70 (0.54 to 0.89)	8.27	0.004
PrEP risk perception (High-level vs. Low-level)	− 0.35 (− 0.69 to − 0.01)	− 1.99	0.71 (0.50 to 0.99)	3.95	0.046

RR: relative risk; CI: confidence interval; HIV: human immunodeficiency virus; PrEP: pre-exposure prophylaxis

Our team conducted two phases of PrEP studies on MSM population in Western China from 2013 to 2015 and from 2019 to 2021, respectively, in conjunction with the 12th and 13th Five-Year Plans of Action for HIV prevention and control in China conducted by the Ministry of Science and Technology of China. In the first phase of the investigation, our findings confirmed the impact of PrEP risk perception on adherence in the MSM population, which was also validated in a subsequent PrEP study (see Additional file 1). The results of the subsequent study showed that the PrEP risk perception scale had good applicability in the MSM population (Cronbach's alpha = 0.795). Based on the GMM, the PrEP risk perception of MSM population was divided into two categories, a high-risk perception group (242/446, 54.26%) and a low-risk perception group (204/446, 45.74%). The parameters indicated a good model fit (Entropy = 0.722, VLRT = 0.002, BLRT < 0.001). According to the GEE, we obtained that PrEP risk perception and adherence were significantly correlated, and high levels of PrEP risk perception hindered adherence (RR = 0.69, 95% CI 0.49 to 0.97, p = 0.036). The results of the two phases of the study remained consistent, indicating the stability and reliability of the findings.

## Discussion

### Trajectory of PrEP risk perception

Until now, few studies have been reported on both PrEP risk perception and its measurement scales. Meanwhile, GMM has been widely used in various disciplines in recent years, such as trajectories of BMI and incident diabetes [30], hypertension [31] and brief psychiatric rating scale [32]. Therefore, we applied GMM to classify PrEP risk perceptions and explain the variability and characteristics of changes in different groups of individuals.

Based on the results of the study, PrEP risk perceptions were divided into a "high-risk perception group" and a "low-risk perception group". Of the 626 MSM, 78.75% had a low PrEP risk perception, indicating that the majority of MSM had a high level of acceptance and uptake of PrEP. According to the temporal trajectory, their PrEP risk perceptions are generally stable and do not change significantly over time. In contrast, for the MSM population with high PrEP risk perception, their risk perception was higher in the early stage. However, there was a significant declining trend, especially in the middle and late stages (The same result was obtained in the subsequent study, with a significant downward trend in the high-risk perception group during the follow-up period). This might indicate that HIV-related health services (e.g., HIV counseling and testing, health promotion, etc.) have a positive effect on PrEP risk perception during follow-up period. Another possibility might be that PrEP users feel more confident that PrEP is working, and become more confident telling others about it and taking the medicine over time. Although PrEP risk perception in the MSM population is a subjective psychological effect, it might be controlled by implementing appropriate measures.

### PrEP risk perception and adherence

A causal relationship between PrEP risk perception and adherence may cannot be demonstrated in observational studies, but longitudinal studies allow the relationship between variables to be determined, which provides a prerequisite for determining causality.

PrEP, as an effective bio-preventive measure, has been shown to be an effective way to prevent HIV infection in high-risk populations. However, its efficacy dependent on high levels of adherence. Previous studies have pointed that risk perception is one of the factors that influence individuals' willingness and motivation [33], which refers to people's feelings, awareness and understanding of risky things and risk characteristics [34]. The PrEP risk perception is an important factor influencing adherence, which in turn is a key component of PrEP efficacy. This demonstrates the importance of PrEP risk perception for MSM PrEP use. Currently, there are few studies on PrEP risk perception and behavior. However, through our analysis, a causal relationship between PrEP risk perception and adherence has been confirmed. Meanwhile, the reliability of the findings was also demonstrated in the subsequent study. According to the results of a longitudinal, qualitative study, low perceived efficacy to adherence was a barrier to PrEP adoption [35]. It indicates that while studying PrEP adherence in the MSM population, we should include not only adherence but also whether the individuals are conscious of the importance of adherence. The results of our study showed that individuals with low perceived risk of PrEP had higher adherence. They are more likely to accept PrEP strategy, and are better able to feel the positive effects while achieving high adherence, thus creating a virtuous circle. It also demonstrates the need of paying attention to PrEP risk perception in this population, which helps to form a better and effective behavior system to improve adherence and preventive effectiveness. In the future PrEP implementation process, while we focus on the PrEP risk perception in the MSM population, we should also emphasize to them the importance of adherence, so that they can fully understand and feel the benefits of forming good adherence, which will help reduce their PrEP risk perception and improve adherence.

### Other factors and adherence

Our findings suggest that the MSM population in the event-driven group had higher adherence, which is consistent with Wu’s results [23]. In addition, MSM who had high-risk anal sex had lower adherence compared to MSM who did not. In fact, previous findings have been already shown that changes in sexual behavior from PrEP will affect the acceptance and use of PrEP [36]. We believe that there are two main reasons.

Firstly, many studies point out that unprotected sex is a manifestation of intimacy [37, 38]. Some MSM regard condomless sex with their primary partner as a way to express commitment, trust and love [39, 40], which let them feel more excitement and pleasure. Kristi [37] refers to these motivation as "intimacy motivation", which is significantly correlated with PrEP adoption intentions. That is, MSM are likely to engage in more unprotected sex for intimacy and hence use PrEP, but in fact their adherence is not high.

Secondly, MSM may have high-risk anal sex as a result of their lack of HIV knowledge understanding. According to the Theory of Knowledge-Attitude-Practice, knowledge is the foundation of behavior change, and is also the strongest predictor of behavior change [41]. At the same time, knowledge about HIV remains a critical element in psychosocial models of HIV risk behavior and is commonly used as an outcome in HIV prevention interventions. A potential relationship between condomless anal sex and HIV knowledge has been demonstrated [42]. Also, our findings showed that a high level of HIV knowledge also positively influences PrEP drug adherence. This implies that knowledge plays an important role in risky sexual behavior and adherence in the MSM population. Insufficient HIV-related knowledge in this population has resulted in the high-risk sexual behaviors and poor adherence.

Based on the two reasons above, the relationship between high-risk anal sex and adherence during PrEP in MSM is explained. In future studies, we should pay more attention to the motivation of PrEP. HIV knowledge should be correctly identified and improved to increase adherence for this population.

### PrEP risk perception scale

In this study, we constructed the PrEP risk perception scale for the first time, based on the dimensions that might influence risk perception. The scale has 16 items based on the 5-point Likert measure, with a total score ranging from 16 to 80. At the beginning, we didn't specify the range for PrEP high risk perception and low risk perception. We believe that providing direct ranges based on descriptive data without statistical calculations is inaccurate. For example, specifying high and low risk perceptions using more than 80% of the overall score. Meanwhile, it was more difficult to directly define high and low levels, because our study was a prospective cohort study with multiple repeated measurements of PrEP risk perception. Therefore, we used a statistical analysis to classify the level of PrEP risk perception based on the parameter estimation of the model. Considering the applicability of the PrEP risk perception scale in the future, more research is needed to prove it. In addition, the dimensions that influence PrEP risk perception will be explored in greater depth, such as conducting qualitative interviews and expert consultations.

As the first longitudinal study to explore the PrEP risk perception in MSM population, we still have several limitations. (1) The sampling method had to be non-probability sampling due to the specificity and invisibility of this population. Also, to construct and model a longitudinal cohort of PrEP risk perception, we screened subjects with ≥ 3 total follow-up visits. It is undeniable that we cannot avoid the possible impact of the screening of other variables on the outcome indicators. (2) This study used a self-administered scale to measure the PrEP risk perception. Although the reliability of its scale was good, it did not always reflect the actual risk of the population. Future studies should continue to explore risk perception measurement tools with higher accuracy. (3) Adherence we used the self-report method, which may lead to overestimate due to recall bias and social desirability bias. (4) The questionnaire covered many sensitive issues, such as sexual behavior, sexual partners, and condom use. Since we use a face-to-face follow-up survey, subjects may not fill out the questionnaire truthfully due to privacy concerns. (5) We combined the time-driven and event-driven groups for the medicine group when conducting the analysis. However, when performing the generalized estimating equations analysis, we included this factor for correction, minimizing the effect on the study results.

## Conclusion

We constructed the PrEP risk perception scale, measured the longitudinal trajectory of risk perception during PrEP in MSM population, and explored the relationship between different levels of PrEP risk perception and adherence. High levels of PrEP risk perception in the MSM population can hinder adherence. The results of this study were validated in the subsequent PrEP projects conducted in 2019 to 2021, which demonstrates the stability and reliability of our findings. This provides a theoretical basis for future research on "risk perception-adherence" to better understand the psychological and behavioral characteristics of this population in the PrEP process, so that it can be improved and optimized to prevent and control HIV transmission. Meanwhile, based on the longitudinal trajectory, PrEP risk perception can be controlled to the desired level if appropriate efforts are taken to improve PrEP effectiveness. In addition, paying attention to the sexual behavior and HIV knowledge of the high-risk population, as well as understanding the motivation of PrEP can also help improve adherence.

## Supplementary Information

**Additional file 1. ** PrEP risk perception and adherence among men who have sex with men: a prospective cohort study based on growth mixture model.

## Data Availability

Because MSM population are very sensitive and private, the data sets used and analyzed in this study are available from the corresponding author upon reasonable request.

## Abbreviations

PrEP: Pre-exposure prophylaxis
MSM: Men who have sex with men
GMM: Growth mixture model
GEE: Generalized estimating equations
RR: Relative risk
CI: Confidence interval
UNAIDS: The Joint United Nations Program on HIV and AIDS
HIV: Human immunodeficiency virus
AIDS: Acquired immune deficiency syndrome
NGOs: Non-governmental organizations
TDF: Tenofovir disoproxil fumarate
EFA: Exploratory Factor Analysis
CFA: Confirmatory Factor Analysis
RMSEA: Root mean-square error of approximation
CFI: Comparative fit index
TLI: Tucker-Lewis index
SRMR: Standardized root mean squared residual
AIC: Akaike
BIC: Bayesian
aBIC: Sample-Size Adjusted BIC
VLRT: Vuong-Lo-Mendell-Rubin Likelihood Ratio Test
BLRT: Bootstrapped Likelihood Ratio Test
