# Supplementary material for: PrEP risk perception and adherence among men who have sex with men: a prospective cohort study based on growth mixture model
Source: BMC Infect Dis. 2022 Dec 30;22:969. doi: 10.1186/s12879-022-07966-3 (PMC9805001; doi:10.1186/s12879-022-07966-3)
Supplement: Supplementary file 1 — Additional file 1. PrEP risk perception and adherence among men who have sex with men: a prospective cohort study based on growth mixture model. [file 12879_2022_7966_MOESM1_ESM.docx]

**1. Introduction of the subsequent study**

In the second phase of the investigation, data on adherence and PrEP risk perception among MSM population were obtained from the National Key Project on Infectious Diseases of the Ministry of Science and Technology of China in the 13th Five-Year Plan (registration number: ChiCTR1900026414, date of registration: 08/10/2019).

The project is based on the intelligent reminder system that aims to improve PrEP drug adherence and reduce new HIV infections in the MSM population. Recruitment methods and inclusion and exclusion criteria for the MSM population were the same as in the first phase of the investigation. The MSM population meeting the criteria was randomly divided into the reminder group, no-reminder group and blank control group. Both the reminder group and the no-reminder group belong to the medicine group. Subjects received daily oral dose of Lamivudine and Tenofovir Disoproxil Fumarate tablets (300 mg/tablet), and followed face-to-face every 12 weeks to complete a new round of follow-up questionnaires. The content of the questionnaire is basically consistent with the previous investigation. The study population screening process is shown in **Figure S1**.


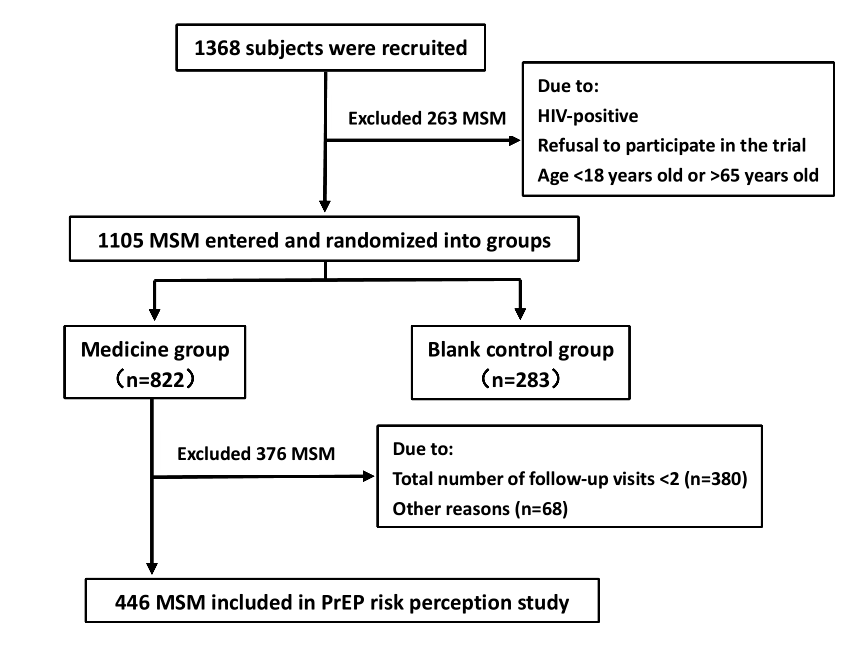


**Figure S1.** Flow chart f of participants’ enrollments.

MSM: men who have sex with men; HIV: human immunodeficiency virus.

**2. Validation of the PrEP risk perception scale**

In the subsequent study, we similarly performed Exploratory Factor Analysis and Confirmatory Factor Analysis on the PrEP risk perception scale. The results of the Exploratory Factor Analysis are shown in **Table S1**. The results of the Confirmatory Factor Analysis are as follows: Chi-square=478.963, df=98, RMSEA=0.072, CFI=0.918, TLI=0.899, SRMR=0.049. Factor Analysis showed the applicability of the PrEP risk perception scale in the MSM population.

**Table S1** Exploratory Factor Analysis of the PrEP risk perception scale

| Factors | Items | Range | Geomin Rotated Loadings |
| --- | --- | --- | --- |
| Personal experience and self-efficacy | I think medicine make me safe, away from AIDS ^a^ | 1-5 | 0.685 |
|  | I can remember to take my medicine on time ^a^ | 1-5 | 0.644 |
|  | My fear of AIDS is lessened ^a^ | 1-5 | 0.815 |
|  | I'm used to taking medicine ^a^ | 1-5 | 0.428 |
|  | | | Cronbach's alpha=0.774 |
| Potential concerns | I worry that the medicine has no effect | 1-5 | 0.415 |
|  | I worry about the side effects of medicine | 1-5 | 0.541 |
|  | I'm worried that homosexual partners know I'm taking medicine | 1-5 | 0.801 |
|  | I worry that other people will discriminate me when they know I am on medicine | 1-5 | 0.758 |
|  | I felt the side effects of the medicine | 1-5 | 0.362 |
|  | I find it inconvenient to take the medicine | 1-5 | 0.358 |
|  | I think it’s very troublesome to take the medicine | 1-5 | 0.336 |
|  | | | Cronbach's alpha=0.845 |
| Medicine impacts | I find it difficult to swallow medicine | 1-5 | 0.686 |
|  | The smell of medicine makes me feel uncomfortable | 1-5 | 0.870 |
|  | The dosage form of medicine I do not like to take | 1-5 | 0.772 |
|  | | | Cronbach's alpha=0.828 |
| Medical mistrust | I think the doctors here discriminate against me ^a^ | 1-5 | 0.881 |
|  | I do not trust the doctors here ^a^ | 1-5 | 0.567 |
|  | | | Cronbach's alpha=0.761 |
| Composite score | | 16-80 | Cronbach's alpha=0.795 |

a: indicates reverse items.

AIDS: Acquired Immune Deficiency Syndrome.

Model fit information of Exploratory Factor Analysis: Chi-square=203.213, df=62, RMSEA=0.055, CFI=0.956, TLI=0.916, SRMR=0.025.

**3. Classification of PrEP risk perception**

Based on Mplus software, GMM was used to classify the longitudinal data of PrEP risk perception of MSM population into latent categories. The model parameters for categories 1, 2, 3, and 4 are shown in **Table S2**. The two classifications of PrEP risk perception had the largest values of Entropy and the p-values of both VLRT and BLRT were statistically significant; therefore, this classification scheme was chosen as the most reasonable. Figure of the two classifications was shown in **Figure S2**. The red line represents “Category 1” (n=204, 45.74%), with a low initial value of PrEP risk perception and a smoother overall level. We named “Category 1” as the “low-risk perception group”. The blue line represents “Category 2” (n=242, 54.26%), with higher initial values of PrEP risk perception and a gradual decrease over time. We named “Category 2” as the “high-risk perception group”.

**Table S2** Growth mixture model (GMM) model classification fit information for each latent category

| Category | AIC | BIC | aBIC | Entropy | VLRT | BLRT | Category Probability |
| --- | --- | --- | --- | --- | --- | --- | --- |
| 1C | 8891.557 | 8957.162 | 8906.384 | - | - | - | 1 |
| **2C** | **8867.625** | **8945.531** | **8885.233** | **0.722** | **0.002** | **<0.001** | **0.4574/0.5426** |
| 3C | 8850.475 | 8940.682 | 8870.863 | 0.622 | 0.088 | 0.007 | 0.4574/0.0112/0.5314 |
| 4C | 8848.670 | 8951.178 | 8871.839 | 0.601 | 0.822 | 0.250 | 0.3300/0.0245/0.1747/0.4708 |

AIC: Akaike, BIC: Bayesian, aBIC: Sample-Size Adjusted BIC, VLRT: Vuong-Lo-Mendell-Rubin Likelihood Ratio Test, BLRT: Bootstrapped Likelihood Ratio Test.

**
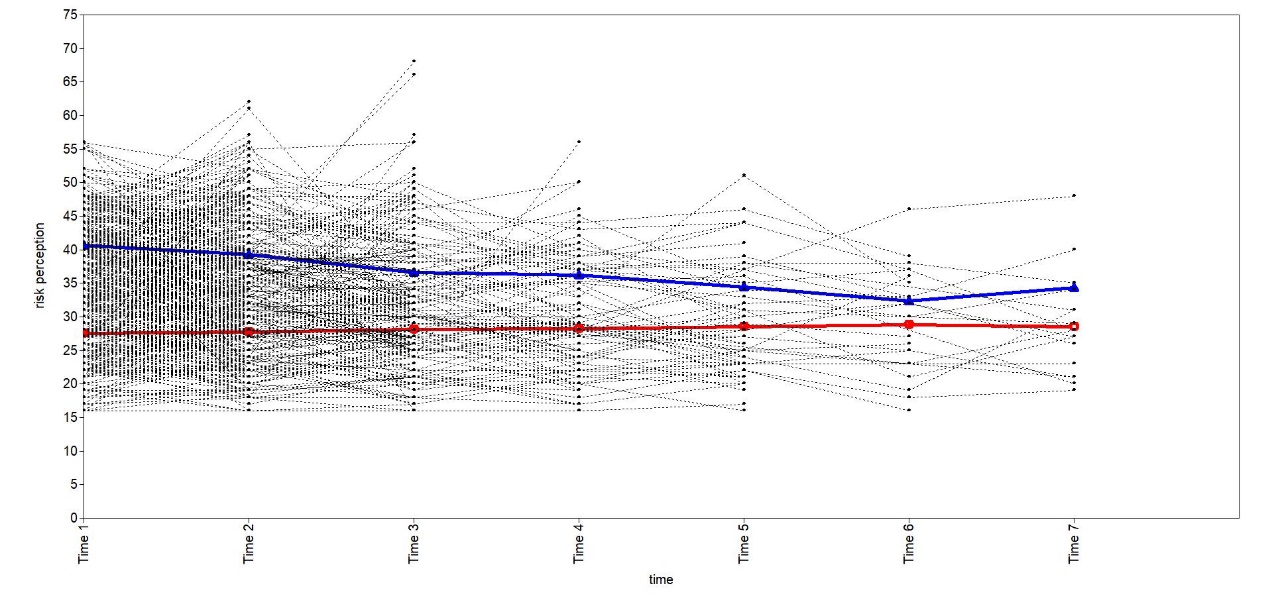
**

**Figure S2.** Trajectory analysis plot of PrEP risk perception two categories in MSM population (we selected). The horizontal coordinate is the time of follow-up, and the vertical coordinate is the score of the PrEP risk perception.

The red line represents “Category 1” (n=204, 45.74%), with a low initial value of PrEP risk perception and a smoother overall level. We named “Category 1” as the “low-risk perception group”. The blue line represents “Category 2” (n=242, 54.26%), with higher initial values of PrEP risk perception and a gradual decrease over time. We named “Category 2” as the “high-risk perception group”.

The GMM model parameter metrics for both categories are shown in **Table S3**. The intercept factor (I) and slope factor (S) variance estimates were 5.739 and -0.444, respectively, with p=0.049 for the intercept factor and p=0.081 for the slope factor, indicating that the initial values of PrEP risk perceptions differed between individuals in the two categories, and the rate of PrEP risk perception growth was also statistically different between individuals. The correlation coefficient between the intercept growth factor and the slope growth factor was 5.284 (p<0.001), suggesting significant correlation between the rate of change in growth and the initial state.

**Table S3** Indicators of the Growth mixture model (GMM) with two categories

| Variables | | | Estimate | SE | Est/SE | p-value |
| --- | --- | --- | --- | --- | --- | --- |
| Category 1 | Mean | I | 27.521 | 0.518 | 53.084 | <0.001 |
|  |  | S | 0.209 | 0.160 | 1.304 | 0.192 |
| Category 2 | Mean | I | 40.675 | 0.542 | 75.016 | <0.001 |
|  |  | S | -1.355 | 0.256 | -5.294 | <0.001 |
| Category 1 vs. Category 2 | Variance | I | 5.739 | 3.158 | 1.818 | 0.049 |
|  |  | S | -0.444 | 0.188 | -2.365 | 0.018 |
|  |  | S With I | 5.284 | 1.245 | 4.243 | <0.001 |

I: intercept; S: slope; SE: standard error; Est: estimate.

We described demographic and HIV-related characteristics for both groups. At the same time, the average value of multiple measurement adherence was taken to compare the difference in the proportion of high adherence between the two groups. According to the Chi-square test results, the high adherence proportions of low-risk perception group and high-risk perception group were 59.80% and 47.93% respectively (χ^2^=6.27, p=0.012), which suggested that the high level of PrEP risk perception had a significant effect on drug adherence and further analysis was required (**Table S4**).

**Table S4** Descriptive analysis of demographic and HIV-related characteristics and drug adherence in low-risk perception group and high-risk perception group

| Variables | All  N=446(%) | Low-risk perception group  N=204(%) | High-risk perception group  N=242(%) |
| --- | --- | --- | --- |
| Demographic characteristics |  |  |  |
| Age |  |  |  |
| Median (IQR) | 33(28~40) | 34(28~39) | 33(27~40) |
| 18~30 | 147(32.96) | 57(27.94) | 90(37.19) |
| 30~45 | 226(50.67) | 115(56.37) | 111(45.87) |
| ≥45 | 73(16.37) | 32(15.69) | 41(16.94) |
| Household register location ^a^ |  |  |  |
| Urban | 325(73.53) | 157(77.72) | 168(70.00) |
| Rural | 117(26.47) | 45(22.28) | 72(30.00) |
| Ethnic |  |  |  |
| Ethnic Han | 413(92.60) | 189(92.65) | 224(92.56) |
| Ethnic minorities | 33(7.40) | 15(7.35) | 18(7.44) |
| Education attainment |  |  |  |
| Junior high and below | 4(0.90) | 0(0.00) | 4(1.65) |
| High school | 23(5.16) | 10(4.90) | 13(5.37) |
| College | 90(20.18) | 40(19.61) | 50(20.66) |
| Undergraduate and higher | 329(73.76) | 154(75.49) | 175(72.32) |
| Marital status |  |  |  |
| Single | 383(85.87) | 180(88.24) | 203(83.88) |
| Married | 63(14.13) | 24(11.76) | 39(16.12) |
| Employment status |  |  |  |
| Employed | 378(84.75) | 172(84.31) | 206(85.12) |
| Internal student | 32(7.17) | 17(8.34) | 15(6.20) |
| Jobless/Retirement | 36(8.08) | 15(7.35) | 21(8.68) |
| Monthly personal income ^a^ |  |  |  |
| <3000 CNY | 102(22.92) | 43(21.08) | 59(24.48) |
| 3000~10000 CNY | 311(69.89) | 145(71.08) | 166(68.88) |
| ≥10000 CNY | 32(7.19) | 16(7.84) | 16(6.64) |
| HIV-related characteristics |  |  |  |
| HIV knowledge score |  |  |  |
| ≥11 | 243(54.48) | 116(56.86) | 127(52.48) |
| <11 | 203(45.52) | 88(43.14) | 115(47.52) |
| Have you been tested for HIV before this time ^a^ |  |  |  |
| Yes | 417(94.34) | 187(92.57) | 230(95.83) |
| No | 25(5.66) | 15(7.43) | 10(4.17) |
| Have you ever received HIV counseling before this time |  |  |  |
| Yes | 323(72.42) | 151(74.02) | 172(71.07) |
| No | 123(27.58) | 53(25.98) | 70(28.93) |
| Sexual role during sexual intercourse with a male partner ^a^ |  |  |  |
| Bottle | 69(18.11) | 31(17.51) | 38(18.63) |
| Both | 89(23.36) | 40(22.60) | 49(24.02) |
| Top | 223(58.53) | 106(59.89) | 117(57.35) |
| Have you had a female sexual partner in the last 6 months (steady and casual) ^a^ |  |  |  |
| No | 370(90.02) | 180(93.75) | 190(86.76) |
| Yes | 41(9.98) | 12(6.25) | 29(13.24) |
| Drug adherence ^b^ |  |  |  |
| Adherence |  |  |  |
| High(≥80%) | 238(53.36) | 122(59.80) | 116(47.93) |
| Low (<80%) | 208(46.64) | 82(40.20) | 126(52.07) |
| Medicine group |  |  |  |
| Reminder group | 234(52.47) | 109(53.43) | 125(51.65) |
| No-reminder group | 212(47.53) | 95(46.57) | 117(48.35) |

a: indicates missing data.

b: drug adherence was measured longitudinally, where adherence was averaged over multiple measurements.

IQR: inter quartile range; HIV: human immunodeficiency virus. CNY: Chinese Yuan Renminbi.

**4. Effect of PrEP risk perception on adherence**

Generalized estimating equations (GEE) were utilized to explore the effect of different levels of PrEP risk perception on adherence by incorporating demographic and HIV-related characteristics (**Table S5**). Finally, we obtained that PrEP risk perception was one of the influencing factors for drug adherence (p=0.036). The higher the PrEP risk perception in MSM population is, the lower their drug adherence become (RR=0.69, 95% CI: 0.49 to 0.97). Also, HIV counseling had a positive effect on adherence (RR=1.58, 95% CI: 1.07 to 2.36, p=0.022). There was no statistical difference in adherence between the reminder and no-reminder groups (RR=1.34, 95% CI: 0.96 to 1.89, p=0.084). The results of the two phases of the study remained consistent, indicating the stability and reliability of the findings.

**Table S5** Generalized estimating equations (GEE) analysis affecting PrEP medication adherence

| Variables | β (95%CI) | Z | RR (95%CI) | χ^2^ | p-value |
| --- | --- | --- | --- | --- | --- |
| Medicine group  (Reminder vs. No-reminder) | 0.30  (-0.04 to 0.64) | 1.73 | 1.34  (0.96 to 1.89) | 2.99 | 0.084 |
| HIV counseling  (Yes vs. No) | 0.46  (0.07 to 0.86) | 2.29 | 1.58  (1.07 to 2.36) | 5.22 | 0.022 |
| PrEP risk perception  (High-level vs. Low-level) | -0.37  (-0.71 to -0.02) | -2.09 | 0.69  (0.49 to 0.97) | 4.37 | 0.036 |

RR: relative risk; CI: confidence interval; HIV: human immunodeficiency virus; PrEP: pre-exposure prophylaxis.
